# Supplementary material for: Antioxidant and Anti-Inflammatory Effects of Traditional Medicinal Plants for Urolithiasis: A Scoping Review
Source: Plants (Basel). 2025 Jul 2;14(13):2032. doi: 10.3390/plants14132032 (PMC12252011; doi:10.3390/plants14132032)
Supplement: Supplementary file 1 [file plants-14-02032-s001.zip › plants-3718663-supplementary.pdf]

Table S1. Lithogenic effects of traditional medicinal plants.

Scientific name	Common name	Region	Study design	Lithogenic markers	Toxic doses and adverse effects	Reference
Glechoma longituba (Lamiaceae)	Naki	Beijin, China	Cell lines HK-2 were incubated with CaOx crystals (67 µg/cm^2) and the effect of the Aq extract of the dried aerial part of *Glechoma longituba* (AExGl) was evaluated for 24 hours.<br><br>C (medium)<br>CaOx (crystals: 67 µg/cm^2)<br>CaOx + Potassium citrate at different amounts:<br><br>CaOx + AExGl (0.5 mg/mL)<br><br>CaOx + AExGl (1 mg/mL)<br><br>CaOx + AExGl (2 mg/mL)<br><br>CaOx + AExGl (4 mg/mL)	**CaOx vs**<br>↓ crystal-induced injury (apoptosis)	ND	[68]
Bergenia ligulata (Saxifragaceae)	*Bergenia ligulata*<br><br>*(Saxifragaceae)*	Chandni Chowk, New Delhi, India	Cell lines HK-2 were incubated with Na_2Ox (2 mM) and evaluated the effect of the EtOH extract of plant and rhizome *Bergenia ligulata* (EexBl) for 24 hours.<br><br>C (DMEM medium)	**Ox vs**<br>↓ crystal-induced injury (adhesion)	> 5000 mg/kg BW of mice<br>No behavioral changes and histological examinations	[69]

			Ox (2mM)<br>Ox (2mM) + Cystone (200 µg/mL)<br>Ox (2mM)+ EexBl (200 µg/mL)			
Lygodium japonicum (Lygodiaceae)	*Lygodium japonicum*	Ulsan, Korea	Wistar rats were induced to UL with EG (0.75%) *ad libitum* for 28 days. The animals were divided into the following experimental groups (n = 6 per group). Treatment with EtOH extract of *Lygodi Spora* (EexLS) was administered orally from day 29 to 42.<br><br>C (water)<br>EG for 42 days<br>EG+ distilled water (1 mL)<br>EG + EexLS (400 mg/kg BW)	**In urine**<br>**EG vs**<br>EG + 400 mg/kg /BW<br>↑Volume urinary<br>↑↑ Cit<br>↓↓ Ox<br><br>**EG + 1 mL distilled water vs**<br>EG + 400 mg/kg BW<br>↑ Cit<br><br>**In kidney**<br>**EG vs**<br>EG + 400 mg/kg/BW<br>↓↓ Ca, Ox, Number of calcium deposits<br><br>**EG + 1 mL distilled water vs**<br>EG + 400 mg/kg/BW<br>↓ Ca<br>↓↓ Ox	> 4000 mg/kg BW of rat<br><br>No appreciable behavioral changes observed.	[70]
Citrus limon (Rutaceae)	Lemon peel	China	Wistar rats were induced to UL with EG (75%) *ad libitum* for 30 days. The animals were divided into the following experimental two groups (n = 6 per group). Treatment with aqueous-MeOH extract of lemon peel (MExLP) daily for days 30-50.<br><br>C (water)<br>EG<br>EG + MExLP (100 mg/kg BW)	**In urine**<br>**EG vs**<br>EG + 100<br>↓ Ca,<br>↓↓↓ Ox<br>↑ Cit<br><br>**In kidney**<br>**EG vs**<br>EG + 100<br>↓↓↓ Ca, Ox	> 2000 mg/kg/BW of rat<br>No signs of intoxication	[71]

Xanthium strumarium (Asteraceae)	Common Cocklebur, Donkeybur	India	Wistar rats were induced to UL with EG (75%) and NH_4Cl (1%) in drinking water ad libitum for 14 days and EG alone for other next 14 days. The animals were divided into the following experimental groups (n = 6 per group). Treatment with EtOH aqueous extract of the fruit part of *Xanthium strumarium* (EExXs) was administered orally from 14th- 28th days.<br><br>Control Group: Water<br>EG + NH_4Cl<br>EG + NH_4Cl + Water (vehicle control)<br>EG + cystone (100 mg/kg BW)<br>EG + EExXs (500 mg/kg BW)	**In urine**<br>**EG + NH_4Cl vs**<br>EG + 500 mg kg<br>↓↓Volume urinary, Ox,<br>↑↑ Ca<br>↑ pH<br><br>**In kidney**<br>**EG + NH_4Cl vs**<br>EG + 500 and potassium citrate<br>↓↓ CaOx crystal deposition Score	>1500 mg/kg/BW of mice<br>Morphological changes in tissue color were observed with slight hepatomegaly and splenomegaly; however, no changes in kidney morphology were observed.	[72]
Tribulus terrestris (Zygophyllacea)	Gokharu	Bangalore, India	Wistar rats were induced to UL with EG (0.4%) and NH_4Cl (1%) in drinking water for 15 days and EG (0.4%) from 16th–28th day. The animals were divided into the following experimental groups (n = 6 per group). Treatment with Aq extract of fruit part of *Tribulus terrestris* (AExTt) administered orally from 16th–28th day.<br><br>C (water) EG + NH_4Cl<br>EG + NH_4Cl + Vehicle<br>EG + cystone (750 mg/kg BW)<br>EG + NH_4Cl+ AExTt (75 mg/kg BW)<br>EG + NH_4Cl+ AExTt (225 mg/kg BW)<br>EG + NH_4Cl+ AExTt (750 mg/kg BW)	**In urine**<br>**EG + NH_4Cl vs**<br>EG + NH_4Cl +75<br>↓↓↓ AU<br>EG + NH_4Cl + 225<br>↓↓↓Ca, AU<br><br>EG+ NH_4Cl + 750 and EG + NH_4Cl + Vehicle<br>↓↓↓Ca AU<br>↑↑↑ Mg	>2000 mg/kg BW of rat<br><br>No adverse effects. No gross pathological changes were observed at necropsy of tissues/organs from any rat.	[73]

Differences between groups are shown by p values: one arrow $p < 0.05$; two arrows $p < 0.01$; three arrows $p < 0.001$. UL: urolithiasis; EG: ethylene glycol; NH_4Cl: ammonium chloride; MeOH: methanolic extract; EtOH: ethanolic extract; Aq: aqueous extract. HK-2: human kidney epithelial cell line; Cr: Creatinine; Cit: citrate; Ca: calcium, Ox: oxalate; AU: uric acid. The plant *Glechoma longituba* has no toxicity studies.
